# Risk Assessment and Experimental Light-Balloon Deployment of a Stratospheric Vertical VLF Transmitter

**DOI:** 10.3390/s23031073

**Published:** 2023-01-17

**Authors:** Tomasz Aleksander Miś, Józef Modelski

**Affiliations:** Institute of Radioelectronics and Multimedia Technology, Warsaw University of Technology, 00-665 Warszawa, Poland

**Keywords:** VLF, propagation, stratosphere, aerostat, airborne, vertical, VED

## Abstract

This paper discusses the risks associated with an aerostat-supported stratospheric (unanchored) balloon mission equipped with a long vertical antenna and a very low frequency radio transmitter. The risks have been grouped into four main types and applicable mitigation methods have been presented to provide a sufficient level of safety and reliability to such a balloon mission. An experimental mission consistent with this analysis, based on the described theoretical VLF propagation approach, has been prepared and launched, and is operating at 14.2 kHz with a vertical antenna of a total length of 400 m and a total payload of max. 4 kg. The maximum altitude reached 29,164 m. The experiment’s signal has been registered in numerous locations in Europe; the results are compared with numerical analysis employing a hypothesis of an apparent transmitting frequency decrease with the rise of the transmitter’s altitude. The numerical analysis explains the behavior of the experimental signal and remains generally consistent with the hypothesis.

## 1. Introduction: VLF Emitted from Airborne Sources

Very low frequency radio signals (3–30 kHz, wavelengths 100–10 km) have been used since the 1920s for planetary wireless communication for various means (telegraphic, time signals, frequency standards [1], narrow-bandwidth digital transmissions, remote sensing of various types [2,3], or even in the excitation of the upper layers of the planet’s magnetosphere [4]) which demands the use of elaborated antenna systems in order to keep the emission efficiency at acceptable levels. As the transmitting structure ought to have its dimensions comparable to the emitted wavelength in order to obtain a certain efficiency, numerous designs have been proposed to expand the vertical elements of the radiating structures with triatics which are horizontal wires suspended from surrounding terrain structures [5,6], or by multiplying the number of transmitting towers that are connected with each other at the top by horizontal capacitive wires [7,8]. With the advances in coil technology, the tuning of such antenna systems have become less problematic [9]. The now-elaborated ground structures are improving the emissions of the VLF signals that have become an important part in the financing of the overall investment (installation and maintenance) of a VLF facility [10].

A possible solution to reduce the costs of a terrestrial installation supporting the radiating elements of the antennas is to employ airborne vehicles to either support anchored transmitting wires, or to lift a free-flying, fully airborne transmitting system that is no longer connected to the ground—both these solutions mitigate the use of any kind of masts or towers and greatly reduce the overall costs on the side of the needed equipment. The first method was successfully employed by prof. Adolf Slaby as early as 1897, when airship-supported wires (up to 400 m of length and up to 280 m of altitude) were allowed to establish a telegraphic communication link [11]. A new adaptation of a similar design—an aerostat-supported antenna wire—was proposed in 1989 and later in 1997, with new tuning technology and elaborated ground-plane radials [12,13]. Fully airborne vertical long wire antennas were frequently employed by the zeppelins in the beginning of the 20th century [14]. In 1927, a design describing the parameters of the unanchored aerostat-supported dipole antenna for lower frequencies was proposed by Grover and was later referred to by Burrows [15]. As aviation technology progressed, aircraft-supported wires became a viable option for VLF transmissions, albeit numerous issues regarding the appearance of corona [16] and the overall performance of such horizontal antenna systems had to be investigated. A rotorcraft adaptation of a horizontal magnetic coil antenna has also been introduced [2], although strictly for remote sensing of ground layers. It was found that for the airborne cases, the vertical long wire antennas create overall stronger electric fields [17,18]. Elaborated numerical comparisons between the ground-based vertical and horizontal (aircraft-trailed) antennas have shown that the horizontal antennas are superior to vertical antennas in terms of electric field strength along a pre-defined propagation path and in a few certain cases, the vertical antenna remained almost constantly superior in performance [19]. This setup, although more proficient in emission performance, poses more engineering challenges to be solved due to the behavior of the airborne vehicle employed, and the various properties of the antenna itself (mechanical, electrical, thermal, and operational).

Developing further the 1927 idea of Grover, a type of experiment using a light stratospheric balloon that elevates the transmitting antenna on altitudes comparable with half of the distance between the ground and the lower layer of the ionosphere was proposed in 2018 [20]. It was based on previous light-balloon experiments in similar conditions that investigate the mechanical and operational properties of such a system, and have been carried out since 2014 [21,22]. The basic advantages offered by a complete transmitting system of this kind shall not only consist of the reduction of the overall cost and needed facilities, but also, as the antenna is moved closer to the middle part of the terrestrial waveguide, the signal coverage and its mode composition are expected to improve in terms of mode composition, mode attenuation, and overall signal strength along a given path. In this paper, the solutions and mitigation strategies to issues related to the risks associated with such light-balloon missions are presented and analyzed, as well as the detailed theoretical basis for airborne VLF signal behavior and the experimental flight results of a VLF emitter operating at 14.2 kHz.

## 2. VLF Theory of Propagation from High Altitudes

Very low frequency radio signals emitted in the terrestrial waveguide (the airspace between the ground and the lower layer of the ionosphere) always tend to propagate using subsequent modes of propagation characterized by specific structures of electric and magnetic fields that are dependent on the physical dimensions of the waveguide including the penetration depth of the signal into the ground/water and the altitude of the effective reflective lower layer of the ionosphere [5,23]. Numerical simulations show that the number of modes effectively contributing to the VLF propagation (total VLF electric field on a given propagation path) during daytime approaches 7 discrete modes [24], with the dominating type of mode (nut not limited to) being the TM, or transverse-magnetic [5,25]; for the lower part of the low frequency (LF) band, this number is elevated to 10 [24]. The zeroth mode of propagation, characterized by a simple structure of an electromagnetic wave with a vertical electric field component and a horizontal magnetic field component, is rarely excited—in contrary to many popularized explanations of the VLF propagation [26]; it is more typical for ultra-low-frequency (ULF) and lower bands for VLF to be attenuated rapidly and to contribute, to a small extent, to the total signal strength over long paths. Over distances exceeding a few thousand km, the dominating modes of propagation are modes 1–3; the higher-order modes are attenuated more rapidly than the lower-order ones, and their contribution to the total sum of the propagation modes is gradually decreasing; above 10 thousand km, the single mode of propagation dominates [25].

The excitation of subsequent modes of propagation can be described in a classical way as a function of transmitting frequency and the relation between the emitted wavelength and the effective vertical dimension of the terrestrial waveguide [23]. The latter parameter could be modified in order to reflect the substantial change of the vertical position of the emitter, as will be shown below. For some other approaches, the excitation of modes is directly and fundamentally associated with the position—the height, or altitude—of the emitter inside the waveguide [27].

### 2.1. Mode Magnitudes

An approach presented by Dobrott and Ishimaru [27], relied mainly on the TM modes and the effect of the terrestrial (planetary) magnetic field on the propagation of the VLF signal, has included a formula describing the vertical electric field strength as a superposition of the of subsequent modes:

(1) $$E_{z}=\overset{n_{mod\_max}}{\underset{n_{mod}=0}{\sum }}C_{n_{mod}}e^{-\gamma_{n_{mod}}x}$$

(2) $$C_{n_{mod}}=\frac{\sin\left(\frac{n_{mod}\pi }{h}ALT\right)}{2\pi n_{mod}-\sin\left(2\pi n_{mod}\right)}$$

where *E_z_* [V/m] is the electric field strength along the Z (vertical) axis inside the terrestrial waveguide, *n_mod_* [−] is the number of the propagation mode, *γ*_*n*_*mod*_ is the propagation constant along the X axis (along the surface of the planet), *C*_*n*_*mod*_ is the mode magnitude factor, h [m] is the height of the conducting layer equal to 80 km (in all the approaches presented in this paper) and *ALT* [m] is the altitude of the emitter.

**Figure 1:**
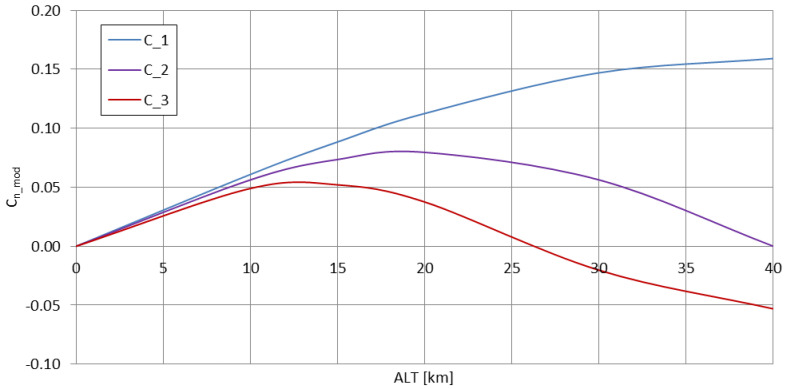
VLF propagation mode magnitudes (1st, 2nd, and 3rd) plotted as a function of the emitter’s altitude.

Formula (2), describing the magnitudes of the subsequent propagation modes, has the altitude included as the main variable. Figure 1 presents these formulas for three main modes of propagation to the maximum altitude of 40 km, which is considered as the half-distance to the ionosphere (for h = 80 km); if the effective lower ionospheric layer is to be considered as a layer having similar physical parameters (e.g., the conductivity) to the lower border of the waveguide, then this value of *ALT* = 40 km could be considered as the altitude of the waveguide’s horizontal symmetry surface.

The presented behaviour of the magnitudes of subsequent modes of VLF propagation shows that the 2nd and 3rd order modes are attenuated much more rapidly than the 1st order mode, and that this behaviour intensifies with the increase of the emitter’s altitude (the <0 values of the 3rd order mode magnitude above 26 km of altitude are purely computational)—it should be therefore expected that for a real high-altitude (stratospheric, >12 km of altitude) application of a VLF transmitting system, the excited signal should be composed of mainly the 1st order mode of propagation, as it is described for the few-thousand-km distances for the earth-bound emitters [25], or for closer distances, but for the emitters operating at much lower frequencies within the VLF band [23].

### 2.2. Mode Excitation Efficiencies

To characterize the facility of excitation of subsequent propagation modes in terms of the radiated power, Watt [5] gives expressions defined as the subsequent mode excitation efficiencies, formulated as the ratios of the radiated power in the given propagation mode to the power radiated in the half space; for the zeroth propagation mode:

(3) $$r_{ground\_mod0}=\frac{P_{\Sigma \_mod0}}{P_{\Sigma \_halfspace}}=\frac{Z_{0}\lambda }{320\pi h}$$

where *Z*_0_ is the free space impedance [Ω], *λ* is the wavelength [m], *h* is the height of the conducting/ionospheric layer [m], *P*_Σ_*mod*0_—power radiated in the zeroth propagation mode [W], *P*_Σ_*halfspace*_—power radiated in the half space around the ground-based radiator.

Comparing a ground-based vertical antenna to the vertical antenna lifted up above the ground (lower conductive layer of the terrestrial waveguide) by the change of the 2π·*ALT*/*λ* ratio [28]—which reaches the values in the order of 10^−^^4^ at *ALT* = 0 and approaches 8 on stratospheric altitudes—the radiated power of the vertical antenna on the ground is ~twice the value of the radiated power of the same antenna in free space [28], which yields an expression for the zeroth mode excitation efficiency for stratospheric altitudes *r*_*strato*_*mod*__0_:

(4) $$r_{strato\_mod0}=\frac{P_{\Sigma \_mod0}}{P_{\Sigma \_full\_space}}=\frac{2Z_{0}\lambda }{320\pi \left(h-ALT\right)}$$

where *ALT* [m] is the radiator’s altitude (as previously) and *P*_Σ_*halfspace*_ [W] is the power radiated by the vertical radiator positioned away from the lower conducting layer.

Similarly, for the *n_mod_* > 1:

(5) $$r_{ground\_mod1+}=\frac{P_{\Sigma \_mode1+}}{P_{\Sigma \_halfspace}}≅\frac{Z_{0}\lambda \cos^{2}\Psi_{n}}{160\pi h}$$

(6) $$\sin\Psi_{n}=\frac{n_{mod}\lambda }{2h}$$

where *P*_Σ_*mode*1+_ [W] is the power radiated in the 1+ modes of propagation and Ψ*_n_* [rad] is the angle between the upward-propagating wave measured in relation to the waveguide’s lower boundary (with the Formula (6) formulated for the assumption/approximation that the waveguide’s walls are perfectly conductive).

Similar to (4), the expression *r*_*strato*_*mod*__1+_ for stratospheric altitudes for 1+ modes of propagation can be formulated as follows:

(7) $$r_{strato\_mod1+}=\frac{P_{\Sigma \_mod1+}}{P_{\Sigma \_full\_space}}≅\frac{2Z_{0}\lambda \cos^{2}\Psi_{n}}{160\pi \left(h-ALT\right)}$$

To analyze the effect presented in Formulas (4) and (7) by *ALT* > 0, a ratio of ‘stratospheric’-to-‘ground’ mode excitation efficiencies can be formulated:

(8) $$\frac{r_{strato\_mod0}}{r_{ground\_mod0}}=\frac{r_{strato\_mod1+}}{r_{ground\_mod1+}}=\frac{r_{strato}}{r_{ground}}=\frac{2h}{\left(h-ALT\right)}$$

The ratio of 1+ mode excitation efficiency to the zeroth mode excitation efficiency in the stratospheric conditions is given as:

(9) $$\frac{r_{strato_{mod1}+}}{r_{strato_{mod0}}}=2\cos^{2}\Psi_{n}=2\left(1-\sin^{2}\Psi_{n}\right)=2-\frac{n_{mod}^{2}\lambda^{2}}{2h^{2}}$$

**Figure 2:**
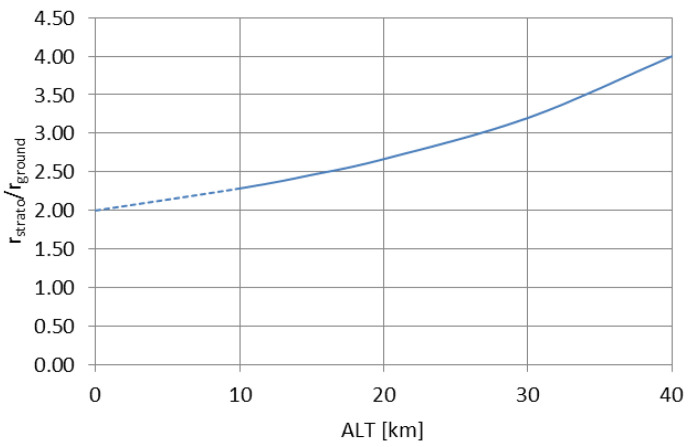
The stratospheric-to-ground mode excitation efficiency ratio plotted for different altitudes.

**Figure 3:**
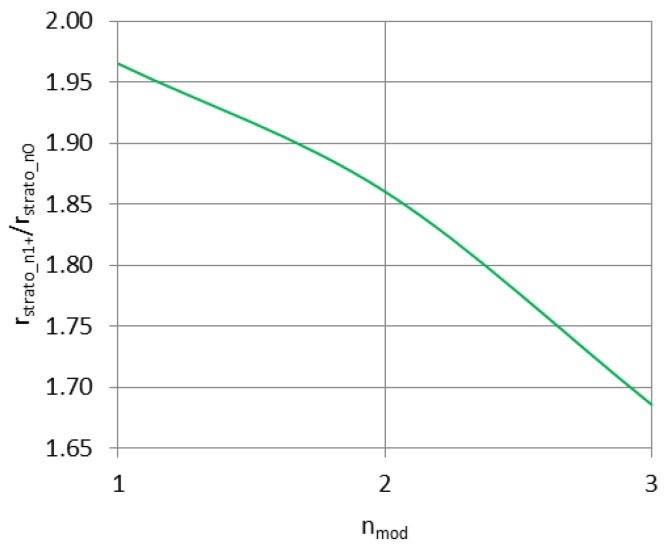
Subsequent mode excitation efficiency ratio for stratospheric conditions plotted for different altitudes.

Figure 2 and Figure 3 present the Formulas (8) and (9) plotted for changing altitude of the radiator, with max. altitude set similarly as in the Figure 1.

The curve in Figure 2 shows that with the rising altitude of the transmitting antenna, the excitation efficiency of the subsequent modes of propagation increases significantly in comparison with the ground-based antenna; for *ALT* = 0 no difference is expected to appear in real conditions (as the stratospheric case becomes equal to the ground case); therefore, in reality the presented curve should begin at the point (0;1) and rise rapidly towards the dashed line to follow the expression (8). In stratospheric conditions, the 1st mode of propagation is excited with nearly twice the efficiency in relation to the zeroth mode, with the subsequent modes (2nd and 3rd) being excited with a gradually decreasing efficiency—a behavior similar to the one presented in Figure 1. Therefore, the most efficiently excited 1st mode of propagation has the greatest magnitude with increasing altitude; the 2nd and 3rd modes of propagation, although excited with only slightly lower efficiency, have their magnitudes decreasing rapidly with the increasing altitude.

### 2.3. Modes’ Attenuation: Hypothesis of Apparent Frequency Decrease

**Figure 4:**
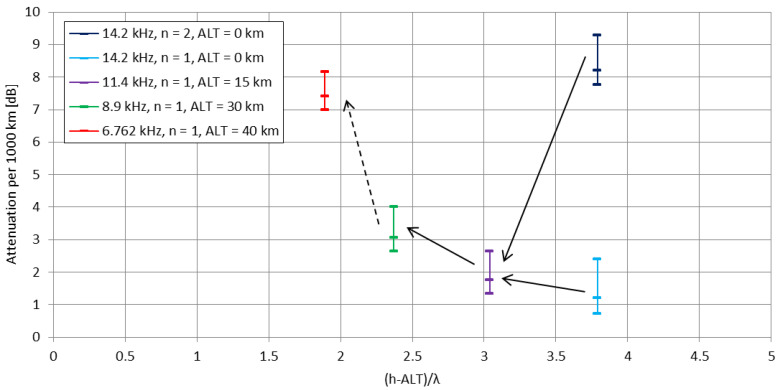
The ranges of propagation modes’ attenuations per 1000 km, plotted for ionospheric height-to-wavelength ratio modified for stratospheric altitudes, presenting ‘modified’ transmitting frequencies. The parameter ‘*n*’ indicates the mode number; the attenuation per 1000 km for each mode has its limits (on the plot: short horizontal markers) depending on the ground conductivity: upper limit for 2 mS/m, lower limit for infinite conductivity, middle value for 20 mS/m. The black arrows indicate the change of modal composition and attenuation for rising altitude; solid black lines indicate altitudes easily reachable by light stratospheric balloons, dashed black line indicates the waveguide symmetry-plane altitude.

The classical paper by Wait [23] that presents the mode theory of VLF propagation shows (ibidem in Figure 3) the computed functions combining the transmitting frequency of the ground-based emitter, the ratio of the height of the conductive layer *h* to the emitted wavelength *λ*, and the attenuation of the signal per 1000 km of distance. Each *h*/*λ* ratio corresponds to its transmitting frequency f. By introducing the expression *h* − *ALT* in place of *h*, as in the Formulas (4) and (7), referring to the changing distance of the airborne emitter to the upper conductive layer of the waveguide (*h* = 80 km), a new, ‘modified’ frequency arises for the (*h* − *ALT*)/*λ* ratio. For this new, altitude-depending frequency, the modal composition of the emitted signal changes, presenting different attenuations per 1000 km of the subsequent propagation modes, which directly indicates their existence and overall contribution to the total emitted signal strength. Figure 4 presents the subsequent mode attenuation ranges, plotted as the functions of ‘modified’ frequencies using the original data from the [23] plot.

For ground conditions, a vertical VLF radiator operating on 14.2 kHz (λ = 21,127 m) presents two essential components of its signal—1st and 2nd modes, with the 2nd mode possessing significantly higher attenuation per 1000 km of distance. With the altitude of the emitter rising, the 2nd mode of propagation becomes more highly attenuated, leaving the 1st mode as the most essential component of the signal. With the altitude rising further, the attenuation of the 1st mode of propagation increases; this corresponds to the ‘modified’ frequency more than 2 times lower than in the ground conditions. Combining these observations with the previously described theoretical aspects of the VLF emission from high altitude emitters—the mode excitation efficiencies and mode magnitudes—the overall theoretical assumption is that in stratospheric conditions (>12 km of altitude) the most efficiently excited, with the strongest magnitude and gradually increasing attenuation per 1000 km is the 1st mode of propagation, with the 2nd and 3rd modes of propagation excited less efficiently, with gradually decreasing attenuation per 1000 km and rapidly decreasing mode magnitudes, especially above 20 km of altitude.

Therefore, a hypothesis for high-altitude vertical transmitting VLF sources’ performance can be formed. The increase of the emitter’s altitude, causing the apparent decrease of the signal’s frequency, shall allow the signal—in accordance to its apparently-lowered frequency—to be attenuated less, and to excite a different, more reception-convenient composition of propagation modes in the terrestrial waveguide (resulting, i.e., in higher signal strength and modified positioning of signal’s nulls). Furthermore, the signal’s apparent frequency decrease shall allow the simulation of signal coverage over a given propagation path for a stratospheric vertical emitter case using the computational case for a ground-based emitter, with its transmitting frequency decreased, according to the actual altitude of the stratospheric emitter (according to the mechanism shown in Figure 4).

## 3. Mission Specifications

The elevation of a VLF emitter to altitudes commensurable with the height of the upper conductive layer of the terrestrial waveguide requires the use of a vehicle different than a conventional aircraft—the aircraft, being an aerodyne, requires a substantial horizontal velocity in order to stay airborne, which practically eliminates the use of a vertical antenna (the closes-to-vertical form is observed during turning/maneuvering [19]). The combustion engines of the aircraft shall also experience major difficulties in the region of the atmosphere with low partial pressure of oxygen (the inability to sustain the flame in the combustion chamber), to which practically only rocket motors with fuels and oxidizers are resistant. The ability to keep the antenna in the desired vertical position and to reach the targeted (stratospheric, >12 km) altitudes can be easily provided by aerostats—stratospheric balloons of various types, lifted up by the buoyant force provided by a lighter-than-air gas (helium or hydrogen) [29].

The balloons themselves differ in designs. For larger missions, plastic foil (or superpressure) balloons are employed, having the ability to achieve a buoyant equilibrium on the given altitude and remain afloat for many days, with the total mass of such a balloon’s envelope exceeding hundreds of kilograms [30]. The increase of maximum altitude reached by such balloons is achieved with the implementation of the envelopes made of thinner and more mechanically resistant plastics. Such balloons are even able to reach the altitudes of the lower layers of the ionosphere [31].

In terms of the smaller balloons, the ones that are more convenient for experiments and tests when considering gas costs, handling procedures, and launch site requirements are the latex ones as the gas inside them expands with the lowering pressure at increasing altitudes. The balloon’s volume increases to the point when the burst of the envelope occurs which marks the beginning of the mission’s re-entry phase. For the plastic or superpressure balloons, the re-entry is triggered by the separation of the payload from the balloon, with simultaneous ripping of the envelope in order to release the lifting gas and bring the plastic back to the ground [29]).

The latex—or sounding, meteorological balloons—are divided into three basic legal classes regarding their payload: light, medium, and heavy [32]. The light class balloon missions have a default ability to freely cross country borders and do not require the use of elaborated tracking devices (including radar reflectors), as well as further agreements for the use of the given airspace and keeping in contact with the local flight control. With the increase of the payload above 4 kg, or the increase of the payload’s side surface density above 13 g/cm^2^, the class of the balloon mission increases, thus requiring the activities mentioned above plus a mandatory device allowing an on-demand flight termination by the separation of the payload from the balloon. However, the length of the possible antenna system attached to the balloon’s gondola is not limited (the only requirements are the breaking under minimal force of 230 N and a visibility-enhancing system of colored pennants or being colored in alternate bands of high conspicuity colors). Therefore, for experimental flights including attempts to launch a series of prototypes of a VLF fully airborne vertical balloon system with the capability of reaching stratospheric altitudes, a light sounding balloon constitutes an attractive option.

**Figure 5:**
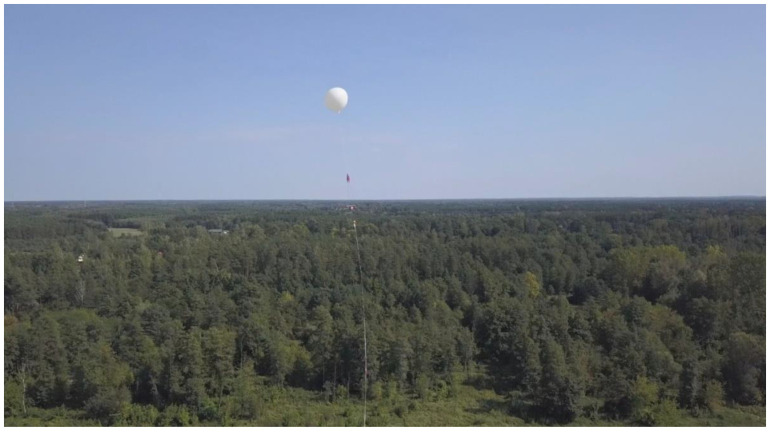
An example of the launch of a light stratospheric balloon mission (12 September 2018) with the deployment of a 40 m-long vertical antenna; seen from the top: the balloon, the parachute, the main gondola, the antenna fixing insulator and the antenna (aluminum tape-form) during deployment.

Since 2014, seven stratospheric balloon flights (light class latex balloons) have been launched from Poland to implement numerous subsystems for the ultimate fully airborne VLF transmitter, as well as to test the procedures that are crucial for a successful deployment, flight, re-entry, and landing of the mission [33]. An exemplary balloon mission with a deploying fully airborne VLF antenna is shown in Figure 5 (4th test flight).

## 4. Risk Assessments

The considered balloon mission, due to its very long payload subjected to highly changing mechanical and thermal environment, can be regarded as highly atypical and, therefore, presenting numerous engineering challenges to be solved in order to put the ultimately designed VLF transmitting system into successful operation. With the challenges, numerous risks are associated that directly affect the performance of the entire experiment and its influence on the external environment (the ground operation, the landing site, and the airspace etc.). To prove that the experiment is safe and able to be performed in the given conditions of a stratospheric balloon flight, the risks have to be grouped into four main types (mechanical, thermal, electrical and operational), and analyzed in terms of their severity and probability. The latter parameters, shown in Table 1, have numerical values assigned which, when multiplied by each other, give the total risk value. The maximum value in this system is 25, which corresponds to the maximum severity with the highest possible probability—such risks in the considered balloon mission case are inexistent. For lower values, equal risk values can be attributed to different situations, e.g., a risk value equal to 8 may indicate ‘damage not affecting performance’ with ‘high’ probability’ or ‘subsystem shutdown’ with ‘low’ probability.

In the next paragraphs, each risk description has its risk value calculated, along with a description of its mitigation (Table 2, Table 3, Table 4 and Table 5). The highest risks are grouped in Table 6 and analyzed further.

### 4.1. Mechanical

The mechanical risks are shown in Table 2. Two out of ten defined risks present a higher than rest risk value. In general, the mitigation of the described issues is related to the correct design of the gondola and the antenna system itself with specific subsystems/solutions tested during dedicated flights [21,33], or employed based on its already-existing space heritage [34,35].

**Table 2:** Mechanical risks for a stratospheric fully airborne VLF light balloon mission.

No.	Description	Probability	Severity	Probability × Severity	Mitigation
M1	Antenna wire break	4	2	8	Division of antenna wire into radiating wire (metal) and supporting tether (poliethylene, multi-thread), with mandatory strength against breaking <230 N
M2	Cable disconnections	4	3	12	Screw connections, cables threaded through circuit boards to remove loads from the solderings
M3	Excessive loads on the mission components	5	2	10	Entire mission designed as a linear structure, with all tethers connected to each other’s ends
M4	Equipment detachment in flight	4	2	8	Additional tethers to external equipment (if needed)
M5	Explosion due to lack of degassing	4	2	8	Avoidance of creation of sealed spaces—addition of small air drains
M6	Early balloon burst	3	2	6	Choice of high-quality balloon
M7	Damages due to balloon burst deceleration	2	3	6	Affixation of components resistant to high accelerations
M8	Damages due to landing deceleration	2	4	8	Affixation of components resistant to high accelerations
M9	Damages due to landing on uneven terrain	2	4	8	Sufficiently thick and shock-absorbing walls of the gondola
M10	Loss of gondola integrity	5	1	5	Temperature- and shock-resistant gondola hatch

### 4.2. Thermal

The list of possible thermal risks is shown in Table 3. Five risks present higher values than the other. The mitigation is, similarly to the mechanical ones, based on either the employment of solutions coming from previous experiments, both from domestic and international balloon missions (choice of materials, glues, insulations etc.), or directly testing them in stratospheric conditions on dedicated flights [21,33]. In the stratospheric environment where the air pressure drops to hundreds of pascals and the external temperature may reach up to −60 degrees Celsius [30,31], the heat transfer phenomenon changes significantly. Instead of convection, the heat can be transferred either by radiation (from an element preferably black and of rough surface) or by conduction over a heat bridge (usually made of copper). Therefore, the low/freezing temperatures are not the only potentially dangerous factors for thermal design. For instance, the significantly lowered ability of a conventional circuit board (e.g., of the navigation unit or of the VLF transmitter) to emit away the heat may lead to thermal overload of the circuit and the loss of its functionality.

**Table 3:** Thermal risks for a stratospheric fully airborne VLF light balloon mission.

No.	Description	Probability	Severity	Probability × Severity	Mitigation
T1	Loss of glue joints performance in freezing temperatures	5	1	5	Use of low-temperature-resistant substances/qualified for space use
T2	Shattering of cable insulation due to freezing temperatures	4	1	4	Exclusion of low-resistant insulations for all insulated cables
T3	Freezing temperature on the frequency generator	4	3	12	Sufficient passive thermal control—surrounding insulation
T4	Freezing temperature on the upper navigation unit	3	3	9	Sufficient passive thermal control—surrounding insulation
T5	Freezing temperature on the lower navigation unit	3	3	9	Use of stratosphere-qualified commercial-off-the-shelf navigation transmitter
T6	Freezing temperature on the battery pack	4	3	12	Sufficient passive thermal control—surrounding insulation
T7	Excessive temperature on the power amplifier	4	4	16	Heat sink attached to the power amplifier’s transistors
T8	Excessive temperature on the power amplifier components	4	4	16	Affixation of heat bridges connected to the main heat sink
T9	Uneven temperature distribution on the transistors	3	3	9	Power amplifier with multiple transistors and load resistors; use of a common heat sink
T10	Fire breakout onboard the gondola	5	1	5	Sufficient electrical insulation on high-voltage and prone-to-overload circuits, efficient heat transfer from hot components
T11	The Joule-Thompson effect during the re-entry phase	4	4	16	Sufficient passive thermal control—surrounding insulation

### 4.3. Electrical

The electrical risks have been grouped in Table 4. They are mostly associated with the changing electrical properties of the atmosphere such as the decrease of the corona- and flashover voltage with the decreasing pressure described by Paschen’s law [36], and the electrification of objects of substantial size that are moving through the electric fields that exist naturally in the atmosphere, especially in the tropospheric region in the clouds [37]. The risk associated with lightning strike has also been included in the highest risks group in this table. Even though this phenomenon is rarely reported [38,39], it bears the highest severity for a light, and highly electrically conductive balloon mission. 

**Table 4:** Electrical risks for a stratospheric fully airborne VLF light balloon mission.

No.	Description	Probability	Severity	Probability × Severity	Mitigation
E1	Short-circuit due to condensed water	4	1	4	Affixation of silica-based desiccants inside the gondola
E2	Excessive electric potential on electronic components due to pyroelectric behaviour of water	4	1	4	Affixation of silica-based desiccants inside the gondola
E3	Corona appearance on the transmitter circuitry	4	2	8	Lacquering of the circuitry, use of lower voltages
E4	Corona apearance on the antenna wire	3	5	15	Corona dischargers concentrating the discharges away from the wire
E5	Transmitter overload due to corona appearance	4	4	16	Automatic detection of overloading with transmitter decoupling/low antenna-transmitter coupling
E6	Transmitter overload due to lightning strike	5	2	10	Automatic detection of overloading with transmitter decoupling/low antenna-transmitter coupling
E7	Flashover on the main antenna insulator towards the main gondola	4	3	12	Proper design of the ‘mushroom’ upper insulator
E8	Interference with other instruments onboard the gondola (near field of the VLF antenna)	3	2	6	Design of the instrumentation within the constraint of operation in the VLF near-field (shieldings, additional filters, digital protocols)
E9	Low stability of the frequency generator	3	3	9	Additional frequency stabilization circuit, passive thermal control around the generator
E10	Loss of power on transmitter subsystems	4	2	8	Division of power source into multiple, separate, independent power sources
E11	Transmitter malfunction (other)	4	2	8	Transmitter ground testing on a dummy load
E12	Transistor gate breakdown	4	1	4	Choice of transistor type with high durability heritage; multiplication of transistors in the power amplifier
E13	Electrical discharge from the system during landing	2	1	2	Corona dischargers concentrating the discharges away from the wire

### 4.4. Operational

Table 5 shows the operational risks defined for the considered experimental stratospheric balloon mission. The very basic aspects of the experiment’s operation have been investigated as early as in 2014 [21], in order to define a pertinent, permanent, and repetitive solution to launch a one-hundred-meter-long antenna directly from the ground. As was mentioned in par. 3, some of the crucial requirements of a safe and visible flight and landing of a balloon mission are also officially covered (as mandated) by the aviation law adopted by the European Union [32].

**Table 5:** Operational risks for a stratospheric fully airborne VLF light balloon mission.

No.	Description	Probability	Severity	Probability × Severity	Mitigation
O1	Incorrect antenna deployment	4	2	8	Elaborated antenna launch procedure
O2	Antenna damage during deployment	4	3	12	Proper choice of launch/deployment site, with sufficient clearance
O3	Air traffic hazard due to antenna length	5	2	10	Affixation of radar reflectors, optical warning systems and a double system of navigation/transponder units (on both ends of the antenna)
O4	Low optical- and radar visibility of the mission	5	2	10	Affixation of large radar reflectors and optical warning signs colored in red or bright orange
O5	Parachute coiling	5	1	5	Sufficiently long parachute tethers
O6	Antenna wire coiling during descent phase	3	3	9	Use of antenna end-weight for movement and re-entry stabilization
O7	Loss of mission tracking	4	2	8	Redundant navigation system
O8	Loss of landing site location	4	2	8	Redundant navigation system, live mission tracking, repeated flight/landing predictions
O9	Landing on water	4	1	4	Positive buoyancy of the gondola
O10	Landing on high-voltage power lines	5	1	5	Antenna wire breaking when subjected to high-voltage short-circuit
O11	Landing on a frequented road	4	1	4	High visibility of the entire flight train
O12	Inflicting damage on external environment when landing	4	1	4	Hard flight train components and main gondola built from/shielded with softened/elastic materials
O13	Reduced amount of delivered RF data due to E5 and E6 risk mitigation	4	3	12	Employment of a large amount of reception points/locations with sensitive receivers and large/ferrite antennas

### 4.5. Analysis of Highest-Grade Risks

Table 5 lists the fourteen highest estimated risks associated with the considered balloon mission. The maximum ‘Probability × Severity’ value in this list is 64% of the maximum value obtainable in this method of risk calculation. All these risks have to be mitigated in order to present the concept of a fully airborne stratospheric VLF transmitter as a feasible experiment.

E4 and E5 risks have been analyzed in detail with the use of experimental data from the domestic balloon missions. The complete reduction of corona is impossible, yet it is possible to estimate and simulate the processes of antenna atmospheric electrification (for verification purposes). In addition, it is feasible to concentrate the discharges in designated areas (spike-like—but soft/pliable—elements of high surface charge density) [40]. It is also possible to manipulate with the discharge voltages using additional sprayed/powdery substances such as talc [41]. The discharges may overload the transmitter’s power amplifier (especially in the E6 risk of the lightning strike). A passive protection against this is the management of the coupling of the transmitter to the antenna; an active solution is one similar to the terrestrial longwave and mediumwave radio stations, where automatic overload detectors connected with ferrite core extinguishers provide rapid protection [42]. However, this solution is too heavy and too dense for a light-balloon mission [41].

T7 and T8 risks are associated with the thermal design approach to the used power amplifier. This has been carried out in [43] using stratospheric experimental data from [44]. Risk T11 is closely associated with the risk M5. To mitigate it, the air intakes need to be located in spaces away from the electronic circuit boards. The M2 risk requires a design approach which includes the methods of cable affixation treated as mandatory in larger balloon missions [34,35]. T3 and T6 risks can be mitigated with the employment of stratosphere-heritage insulations, including the frequently employed Styrodur, a more dense (with less air) version of the popular Styrofoam [44].

The O2 risk was a subject of research in the beginning of the experiments in 2014, with a successful and safe launch procedure ultimately defined [21]. Risk E7, closely associated with E4, has been analyzed in a dedicated work performed using original data and designs from the RCA experiments in VLF technology insulators [45]. Risks O3 and O4, associated with the requirements given by the law, are mitigated easily and effectively by the implementation of solutions already mentioned in the legal document [32]. Risk O13, the direct effect of the mitigation of risks E5 and E6, has been mitigated in a way described in par. 5.1.

Finally, the M3 risk is mitigated by the correct mechanical design of the entire flight train, from the affixation of the balloon to the main tether, to the lower end of the VLF antenna. The flight train shall provide a uniform path for the forces acting on all the tethers and the antenna wire, thereby allowing them to function as a uniform mechanical system with no force-binding joints of lower material strength than the weakest used tether. The system needs to withstand not only the mass forces generated by the payload, but also all aerodynamic forces during its movement, with the possibility of being subjected to oscillations and mechanical standing waves, locally increasing the tensile stress of the tether (as described by e.g., prof. Piccard [46]). Nevertheless, the tether’s maximum tensile strength must allow it to break under the force of 230 N.

The overall analysis of the risks and the possibilities of their mitigation—in many cases possible in multiple ways and solutions—with the favorable provisions of the aviation law adopted by the European Union of, e.g., not limiting the geometric dimensions of the antennas, shall allow the design and execution of tests of fully airborne (not anchored) stratospheric light-balloon VLF transmitters.

**Table 6:** Highest-grade risks for a stratospheric fully airborne VLF light balloon mission.

No.	Description	Probability	Severity	Probability × Severity	Mitigation
E5	Transmitter overload due to corona appearance	4	4	16	Automatic detection of overloading with transmitter decoupling/low antenna-transmitter coupling
T7	Excessive temperature of the power amplifier	4	4	16	Heat sink attached to the power amplifier’s transistors
T8	Excessive temperature of the power amplifier components	4	4	16	Affixation of heat bridges connected to the main heat sink
T11	The Joule-Thompson effect during the re-entry phase	4	4	16	Sufficient passive thermal control—surrounding insulation
E4	Corona appearance on the antenna wire	3	5	15	Corona dischargers concentrating the discharges away from the wire
M2	Cable disconnections	4	3	12	Screw connections, cables threaded through circuit boards to remove loads from the solderings
T3	Freezing temperature on the frequency generator	4	3	12	Sufficient passive thermal control—surrounding insulation
T6	Freezing temperature on the battery pack	4	3	12	Sufficient passive thermal control—surrounding insulation
O2	Antenna damage during deployment	4	3	12	Proper choice of launch/deployment site, with sufficient clearance
E7	Flashover on the main antenna insulator towards the main gondola	4	3	12	Proper design of the ‘mushroom’ upper insulator
O13	Reduced amount of delivered RF data due to E5 and E6 risk mitigation	4	3	12	Employment of a large amount of reception points/locations with sensitive receivers and large/ferrite antennas
O3	Air traffic hazard due to antenna length	5	2	10	Affixation of radar reflectors, optical warning systems and a double system of navigation/transponder units (on both ends of the antenna)
O4	Low optical- and radar visibility of the mission	5	2	10	Affixation of large radar reflectors and optical warning signs colored in red or bright orange
M3	Excessive loads on the mission components	5	2	10	Entire mission designed as a linear structure, with all tethers connected to each other’s ends
E6	Transmitter overload due to lightning strike	5	2	10	Automatic detection of overloading with transmitter decoupling/low antenna-transmitter coupling

## 5. Experimental Deployment

Based on the test flights organized in the previous years (both domestic [21] and international [34,35]), covering numerous aspects of the mission subsystem’s design, a VLF fully airborne antenna system with an operating TX has been prepared as a natural continuation of the chosen experimental approach. However, additional requirements have been issued to the overall system design, as the VLF experiment was to be carried out on the domestic flight train shared with other experiments (UV light intensity measurement and camera test for the monitoring of Transient Luminous Events)—contrary to the fundamental mission design presented in [20], this VLF transmitter was to be integrated within the main gondola positioned typically, i.e., below the parachute. The experiment has been planned for repetitive use in future flights in all weather conditions permitting the balloon launch.

### 5.1. Mission Design

**Figure 6:**
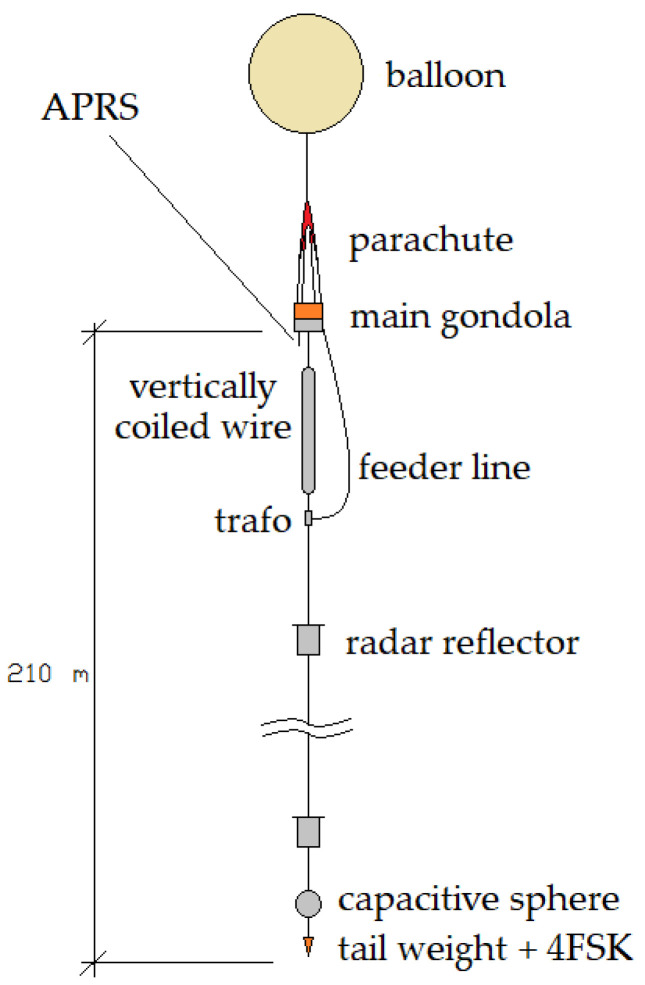
The schematic of the VLF fully airborne VLF transmitting system.

The experimental flight train has been designed in order to fulfill the general requirements defined by the targeted environment of operation (the stratosphere) [31], the main gondola occupation architecture (other experiments onboard), the regulations for the light class of the balloon mission [32], and the regulations for the sub-class no. 36 (mobile service inductive application) of the list of the 1st-class devices according to the European Commission Decision 2000/299/EC (version January 2018) [47]. The overall schematic of the mission is shown in Figure 6.

The main gondola, built from Styrodur and covered in aluminum on the lower half, housed the mission’s experiments (including the VLF transmitter) and the upper APRS navigation transmitter, operating at 144 MHz with the transmitted description ‘14.2 kHz inductive A1′, indicating the transmission type—carrier signal. The antenna system was affixed below the gondola to the tethers directly supporting the parachute; the upper fixing point of the radiating wire was secured with a ‘mushroom’ insulator in order to reduce the electric field intensity in this region [45].

The antenna design followed past experiences from using shortwave meteorological probes with symmetrical antennas [48] and of V. Väisäla’s probes with the antenna system galvanically separated from the transmitter circuit [49]. It consisted of a radiator composed of a schematic wire of five threads in a lacquered painted cotton insulation, supported by a polyethylene tether with affixation points every ~40 m in order to reduce the tensile strain on the wire. The total length of the wire reached 400 m, with the physical length of the system reduced to 210 m by vertically coiling/folding of the upper part of the antenna below its upper fixation point. This contraction allowed the gondola-mounted transmitter to be easily connected to the middle of the antenna’s electric length, although the antenna’s form in full deployment ceased to resemble a classic dipole. The transmitter was connected to the antenna by an insulated twisted-wire feeder line and the air-core antenna transformer that was wound on a metal support connected directly to the antenna wire and the supporting tether. This type of fixation provided the transformer with low total inductive coupling (with winding inductance of 8.56 μH, being approximately 2/3 of its theoretical value due to the magnetic losses generated by its fixation on the wire and tether), capacitive coupling (due to the size of the winding and its support) and its acceptably low mass to be included in a light-balloon experimental flight. The external wire connections were screwed in. The lower part of the antenna was equipped with an aluminum-covered capacitive sphere (400 mm in diameter) to increase the effective length of the antenna system and the tail (end) weight, assuring a safe and stable re-entry phase of the mission. The antenna’s lower end also had a second navigation system affixed—4FSK on 437.6 MHz, in order to indicate the physical end of the fully airborne wire when/if extending over adjacent flight levels.

As mentioned in the previous paragraphs, the chosen operating frequency was 14.2 kHz (wavelength 21,127 m) before 1945 which was attributed to Polish stations AXO/AXL (later SPL) [50,51], now unoccupied in the VLF band. The radiation resistance, calculated for a symmetrical dipole of equal electrical length and effective length of 200 m, reached 0.14142 Ω [52] (short dipole << emitted wavelength); the calculated tuning inductance for this antenna at the given frequency reached 278.013 mH [52], which was divided in half and symmetrically introduced into the upper and lower parts of the antenna with the use of small ferrite-core inductors. The transmitter employed the adapted design previously used in the high-altitude experiments in the IGLUNA program [53,54] (with no modulating transformer), with the power amplifier based on four POWER MOSFET transistors with elaborated heat sinks [43], excited with a switching-circuit-based frequency generator tuned by an RC circuit (which proved to remain acceptably stable in glacier-based field campaign of [54]). The main power source was constituted of a 56-V battery pack which allowed the transmitter to function during the entire flight. The power amplifier operated in class A (linear with 12-V transistor gate bias, similarly as in [53]), with total ohmic resistance of 112.5 Ω.

For safety reasons, the antenna system was equipped with visual pennants and radar reflectors made of aluminized foil, affixed onto horizontal struts made of carbon fiber. To facilitate the recovery of the gondola after landing, the gondola’s side walls were painted in bright orange. The entire mission fit in the light weight class (<4 kg) and was lifted by a Pawan 1200 latex balloon filled with helium (grade 4.6, provided by MESSER Polska, Chorzów, Poland).

From the reception side, a group of VLF listeners was mobilized via the Alexander Association from Sweden (World Heritage Grimeton Radio Station) to assist in the experiment by recording the indicated part of the VLF spectrum and its basic parameters (signal strength, noise level, etc.). The most desired method of signal detection would consist of a series of highly sensitive receivers equal in design and software and spread over a large area (in continental scale). However, this was impossible to physically carry out within the given organizational and financial constraints, as well as with the need of calibration of every individual receiver regarding the local noise level and local interferences (mostly coming from unshielded alternating current machines and high-voltage networks), both constant and ephemeral. The network of separate, highly sensitive receivers operated by skilled individuals in familiar environments provided the best obtainable approach in the planned experiment towards the quality of the reception in the given geographical area by the highest obtainable reduction of interferences and the reduced risk of a trigger by false signal (for a fully automated system, this could pose a significant issue).

### 5.2. Flight Performance

The experiment was carried out on 12 September 2020 from the Center for Aerospace Research of the Warsaw University of Technology (OBLOT PW), airport Przasnysz-Sierakowo (code: EPPZ). Consistent with previously developed launch methods [21], the antenna was deployed from the ground straight into its operational position. The launch (the rising of the antenna) was, however, interrupted by an incidental contact of the antenna’s lower end with the anemometric mast. This caused the separation of the capacitive sphere and the tail weight from the antenna. This incident occurred due to the official yet incorrect (too narrow regarding the changes in wind direction) assignment of launch space on the airfield.

**Figure 7:**
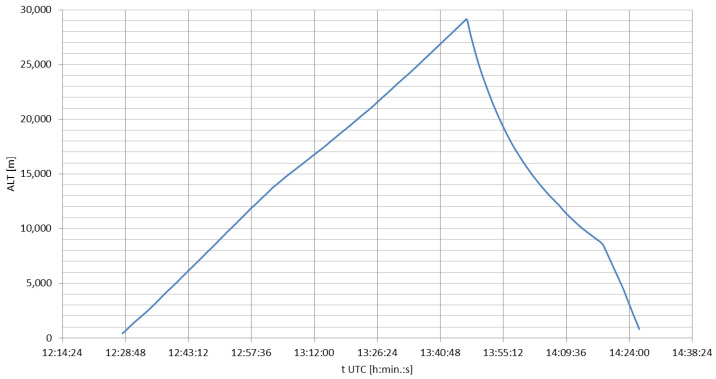
The altitude profile of the experimental fully airborne VLF light-balloon mission from 12 September 2020.

The mission lifted off at 14.28 local time (UTC+2) and touched down at 16.26 local time, 67 km from the launch site, reaching the maximum altitude of 29,164 m above the mean sea level (with the target altitude of 30,000 m) at 15.46 local time and 43 km from the launch site. Figure 7 shows the altitude profile of the mission in time (UTC). The increased loss of altitude at the final phase of the flight is a result of the anemometric mast incident from the launch. The lack of tail weight on the antenna resulted in a total loss of the antenna’s shape during the re-entry phase. At 16.18 local time, the coiled antenna had fallen into the parachute’s tethers and canopy, lowering its aerodynamic drag and increasing its vertical velocity. The mission landed on soft soil in the field and suffered few mechanical breaks, none of which was critical to the main structure of the flight train.

## 6. The Analysis of Flight Results

The listening reports received by e-mail in the upcoming days after the experiment reached 61, with five classified as errors (incorrect time of signal registration etc.), nine unknown results (low quality of the spectrum/FFT etc.), 32 null results (too high noise level, too many interferences etc.) and 14 successful receptions. The useful flight data presented and discussed in the sections below have been registered during the normal operation of the antenna, i.e., with its vertical position, with one hypothetical exception shown in tone of he further Figures. 

### 6.1. Experimental Signal Coverage

**Figure 8:**
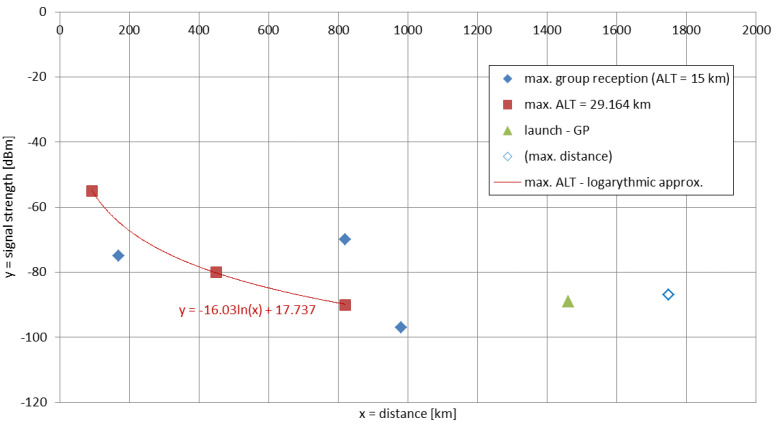
The registered signal levels on different distances from the launch site.

Among the successful receptions, seven delivered enough data to be processed in the analysis. All registered signals were consistent with the emitted signal type, i.e., narrow-band signal consisting of a carrier wave with intensity changing with the altitude and corresponding to the evolving modal superposition. The receiving locations (with approximate distances measured from the launch site—as in continental scale the mission travelled over an insignificant horizontal distance) were: Pruszków (Poland; 93 km), Braniewo (Poland; 170 km), Rivne (Ukraine; 450 km), Forchheim (Germany; 775 km), Salzburg (Austria; 820 km), Wabern (Germany; 820 km), Cologne (Germany; 980 km) and Guingamp (France; 1750 km). Figure 8 presents the recorded signal strengths in these locations. The timing of the receptions showed that the majority of the reports were registered simultaneously at the time when the mission reached ~15 km of altitude. The signals recorded at the time of the maximum altitude over the given distances, could be approximated with a logarithmically fitted function. The value for the 1750 km distance has been included as additional due to the lower quality of the registered information (low quality FFT). The estimated radiated power of the experiment reached ~−30 dBm (~1 μW), which is consistent with the built experimental setup. Figure 8 also lists an additional value obtained from Middlesbrough (UK; 1460 km), which was registered only in the moment of incidental contact of the antenna with the anemometric mast during the launch. Both events are time-consistent; the phenomena can be explained by the fact that during the contact with the mast, the antenna’s effective length increased substantially along with all the underground elements of the mast, allowing the system to function as an anchored vertical long wire antenna.

**Figure 9:**
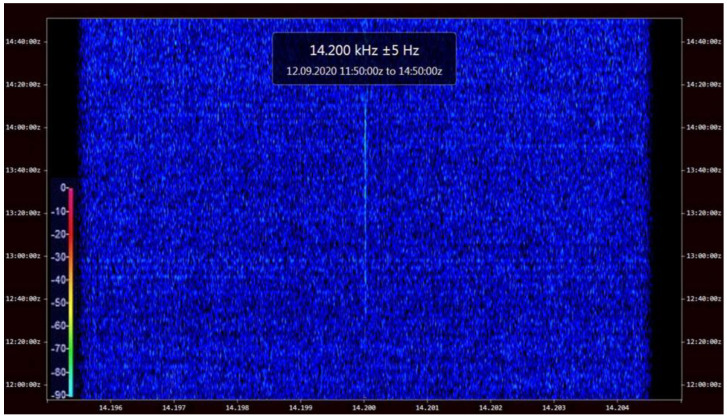
Signal power spectrum (dBm) of the stratospheric VLF light-balloon experiment produced in listening station near Salzburg, Austria (vertical axis: time UTC); courtesy of Christoph Ratzer.

The pattern of signal levels shown in Figure 8 cannot be easily approximated by a simple function, which is consistent and typical for this frequency range, as the signal exhibits many local maximums and minimums (these maximums and minimums may also exist between the interpolated points for the maximum altitude) [5]. A provided exemplary signal spectrum from the entire flight is shown in Figure 9, registered by Christoph Ratzer in a listening center near Salzburg, Austria. The employed NetSDR receiver operated on an external oscillator with the GPS-controlled accuracy [55]. The spectrum clearly shows the maximums and minimums of the signal evolving in time due to the changing composition of the propagation modes with increasing altitude. The minor (in the order of seconds) signal decreases may be produced by a couple of sources such as—the short ionospheric fluctuations [25], and the wind-caused rotation of the main gondola which causes the rotation of the feeder line thus potentially distorting the radiation pattern of the antenna (although the cadence of this movement is high, up to 1 movement per second), or any wind-caused distortions of the radiator’s shape which are at least an order of magnitude shorter than the radiator itself, thereby producing much lower inclination angles than those at which significant differences in signal strengths have been calculated in past research [19]. An increased signal strength on the spectrum can be noticed after the time of the maximum altitude. This is explained by the fact that the flight train had its tail weight severed in the anemometric mast incident, which caused—during the acceleration after the balloon burst (with calculated max. value of −1.56 m/s^2^)—an immediate contraction of the antenna system, resulting in the fall of the antenna’s transformer in the coiled radiating wire, thereby increasing its inductive coupling.

### 6.2. The Evolution of the Antenna Radiation Pattern

As the VLF radio signals possess lower attenuation in different environments—a feature particularly useful in geophysical remote sensing [2]—a difference between the actual boundary and the lower physical surface of the terrestrial waveguide would be expected. The experimental mission travelled above three regions of different ground conductivities of 0.004, 0.008, and 0.015 S/m [56]. The corresponding penetration depths δ [m] can be calculated using a popular formula [57]:

(10) $$\delta =\sqrt{\frac{1}{\pi \sigma f\mu }}\;$$

where *σ* is the ground conductivity [S/m], *f*—the transmitting frequency (here equal to 14,200 Hz) and *μ*—the magnetic permeability, equal to 4π·10^−7^ H/m [58]. For the aforementioned ground conductivities, the penetration depths reached 66.78 m, 47.22 m, and 34.49 m, respectively. These value are incomparable to the total change of altitude of the emitter in this case. The ground conductivities below the emitter also do not contribute directly to the signal strength at the locations of the receptions (a detailed profile of conductivity over a given propagation path would be useful in this case) but may cause a difference in the rising antenna’s radiation pattern. This change—for an approximation of the experimental antenna by a short monopole [28]—can be described by the number *n*, indicating the number of separate lobes created in the radiation pattern due to the process known as ‘scalloping’ due to the changing distance between the antenna and the conducting plane below it [28] (with the variables explained in previous formulas):

(11) $$n=\frac{2\cdot ALT}{\lambda }+1$$

The final normalized power pattern (with *θ* as the directed elevation angle [rad]) can be written as [28]:

(12) $$F\left(\theta \right)={\left[\sin\theta \cos\left(\frac{2\pi \cdot ALT}{\lambda }\cos\theta \right)\right]}^{2}\;$$

**Figure 10:**
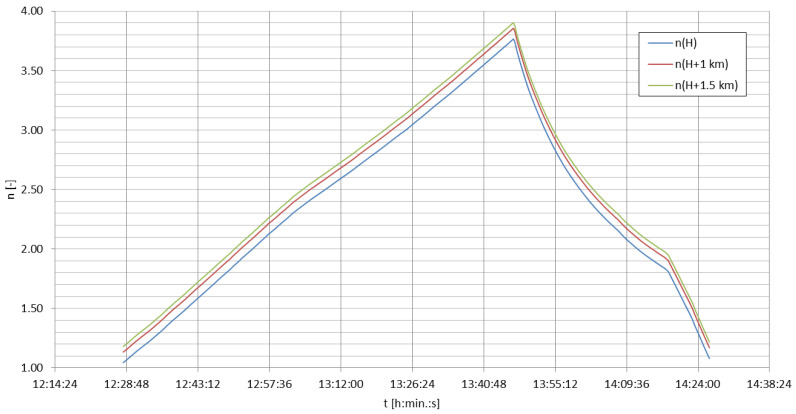
The changing number of lobes of the antenna’s power pattern plotted as a function of the mission’s altitude (time t in UTC). For comparative purposes, two curves for the hypothetical penetration depth (waveguide expansion) of 1 km and 1.5 km have been added. Even for such significant changes (in the order of km instead of m), the number of lobes in the pattern does not change significantly and is always below 4.

**Figure 11:**
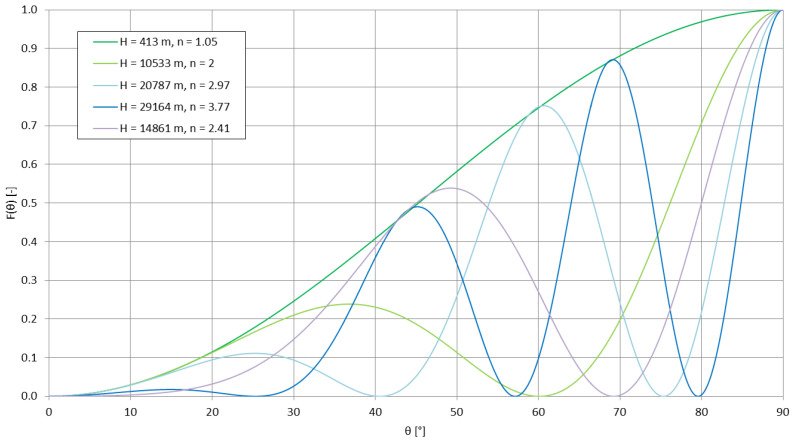
The antenna’s power pattern evolution with the changes of altitude and the respective numbers of lobes. As the pattern is axisymmetric, its cross-section between 0 and 90 degrees is shown. The *n* = 1.05 corresponds to the first altitude indication; for *n* = 2.97 the ground conductivity below the emitter changed from 0.004 to 0.008 S/m; for *n* = 3.77 the maximum altitude was reached; for *n* = 2.41, the ground conductivity below the emitter changed from 0.008 to 0.015 S/m.

**Figure 12:**
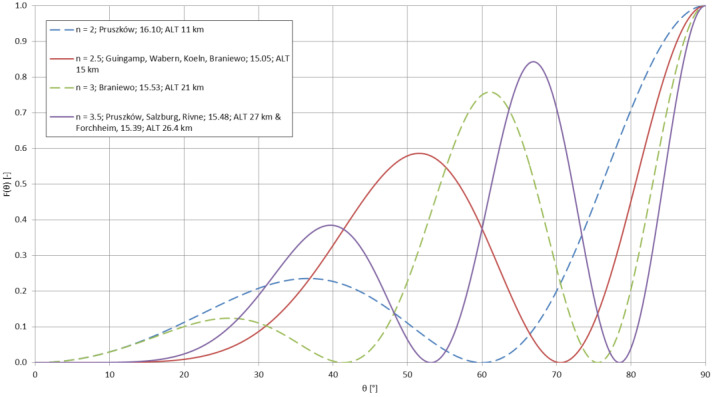
The antenna’s power pattern evolution for the indicated locations of the signal’s reception. The dashed lines represent the hypothetical patterns of the mission during the descent (as the antenna was coiled due to the loss of its tail weight during the launch, the actual pattern was expected to differ from a typical monopole).

**Figure 13:**
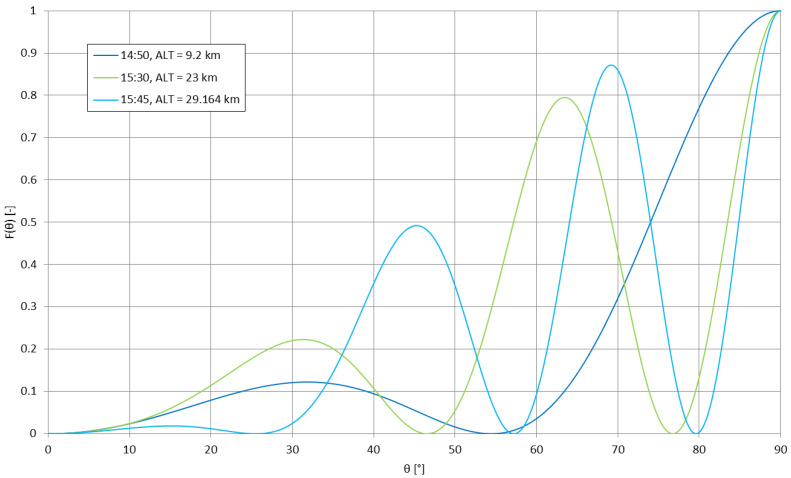
The antenna’s power patterns for maximum signal strengths and their corresponding altitudes, as seen on the spectrum in Figure 9.

Figure 10 shows the change of the number of lobes in the antenna’s power pattern during the duration of the mission. Figure 11 and Figure 12 present the power patterns (12) plotted for different numbers of lobes for different altitudes, corresponding to the characteristic stages of the experimental mission; Figure 13 presents the same power pattern, but strictly in relation to the maximums seen on the power spectrum in Figure 9. It can be seen that the number of lobes is not significantly affected by the changes of ground conductivity below the emitter (as the change of altitude is the leading factor for these changes); the power pattern of the emitter presents intense scalloping, but the total number of lobes in the pattern does not reach 4. The angles of the main lobes of the pattern also change significantly, but this does not indicate that the receptions at the given stages of the mission have been directly related to these angles, as the signal propagation on this frequency inside the terrestrial waveguide is modal [5]. The total superposition of modes in the reception locations could also depend on the antenna’s power pattern’s scalloping.

The maximum directivity of such an antenna is reached for the number of lobes for the altitude *ALT* = 0.4585*λ* [28], which corresponds to the altitude of approximately 9686 m; the Austrian spectrum shows a small increase in signal strength close to the altitude of 9200 m. It would, therefore, be possible for the maximum antenna directivity to contribute to this local maximum, yet it appears not to be the single leading factor in this modal superposition.

### 6.3. Simulated Signal Coverages

A rapid, frequently employed solution to estimate the signal coverage by a ground-based VLF transmitter of given parameters (radiated power, antenna gain) is the Austin-Cohen formula [52], which does not, however, reflect the actual existence of local minimums and maximums in the signal coverage, being that they are products of modal interference that depend on the parameters of the terrestrial waveguide (e.g., the height of the lower ionospheric layer, changing from day to night) [5]. More accurate simulations of the VLF signal propagation require the employment of the modal propagation, performed in dedicated computer software packages. One of these packages is the LWPC (Long Wave Propagation Capability) which enables calculations in the VLF and lower LF frequency ranges over given paths and daytimes (e.g., in [24]), although all are for the ground-based transmitters. Another type of software package is the Longwave Mode Propagator (LMP), originating from the Massachusetts Institute of Technology, and operating in the more accessible Julia programming environment [59]. Having the simulation constraint of the ground-based transmitter, an attempt to calculate the signal coverage was made using the hypothesis given in the Section 2.3 and shown in Figure 4, i.e., the signal’s frequency apparently decreases with the altitude; therefore, it should be possible to simulate an elevated VLF emitter using a ground-based VLF emitter operating on a decreased frequency.

**Figure 14:**
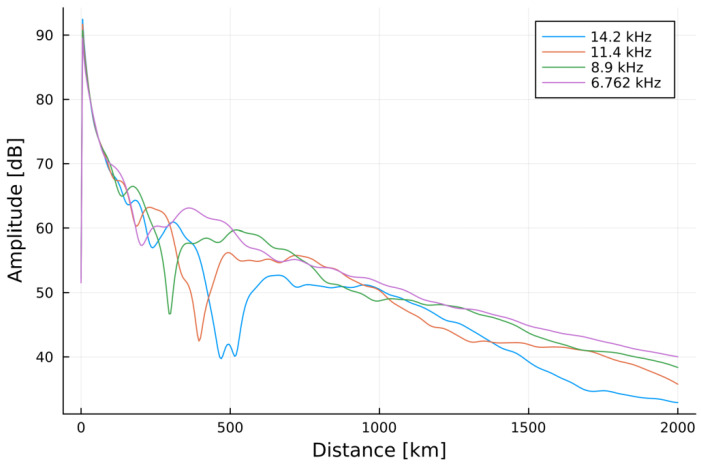
The LMP-simulated signal coverage along the 2000 km path for four VLF frequencies (amplitude in arbitrary units), corresponding—in accordance with Figure 4—to the emitter’s altitudes of 0, 15, 30, and 40 km.

Figure 14 shows the simulations carried out in the LMP software package for the frequencies specified in Figure 4. The transmitter had neither the efficiency, nor the specific radiation patterns indicated (modelled by default as a radiating element) and operated with 1 kW of radiated power (simulation method analogous to those used in the past for broadcasting longwave calculations of the signal coverage [60]) over a propagation path with the ground having the average conductivity of 10^−4^ S/m, and the relative electrical permittivity of 10, height of the lower ionospheric layer of 80 km (as in Figure 4), the planet’s magnetic field strength of 50 μT, and the propagation path sampling rate of 5 km with a maximum range of 2000 km. As the simulations have been carried out for a generalized environment, the plots are expected to present the general tendencies and behavior of the VLF signals along this path, i.e., the approximate positions and movements of the major nulls, the orders/successions of the signal’s amplitude for given distances from the emitter for subsequent frequencies/altitudes of the emitter and the behavior of the signal’s decrease (oscillating or approaching logarithmic).

## 7. Discussion

The most prominent feature of the curves shown in Figure 14 is, for the rising altitude, the change of positioning and the magnitude of the major null of the signal moving closer to the emitter from 500 km at 14.2 kHz/0 km to nearly 220 km at 8.9 kHz/30 km. In numerous regions of the propagation path, the signal also changes its intensities with the emitter’s altitude (and, therefore, with the mission’s time)—a phenomena which is expected to be traceable in the reception reports.

### 7.1. Simulations vs. Experimental Signal Coverage

The majority of the reports with data shown in Figure 8 come from the geographical western part of the continent. The only data positioned to the East from the mission’s trajectory is the Rivne data (Ukraine), which is 450 km away from the EPPZ airport. For the indicated relatively low (for VLF) distance from the emitter, the effect of differentiation between the signal strengths over East-to-West and West-to-East paths is negligible [27]. The Rivne report on the signal strength had explicitly stated that ‘the signal appeared almost at the end of stage of rising of the balloon’ mission. This is consistent with signal evolution in Figure 14, where the 450 km distance initially remains very close to the major null and rises approximately in 15–20 dB at the maximum altitude.

For the Austrian location at the distance of 820 km, the signal strength evolved in a more complicated manner. The rise of the signal strength in the middle altitudes (<30 km) can be found at this distance in Figure 14, as well as the moderate rise of signal strength at the maximum altitude, yet the complete mechanism of the evolution of the signal coverage in this area should include the waveguide dimension changes inflicted by the nearby mountains. At the same distance, but in a different location (Wabern, Germany) at 15 km of the emitter’s altitude, the signal is indicated as higher than for the Salzburg location at maximum altitude. This is consistent with the LMP simulation. In the region of 800-km distances, the differences between the simulated signals at 15 km and 30 km correspond to the differences recorded in the experiment.

**Figure 15:**
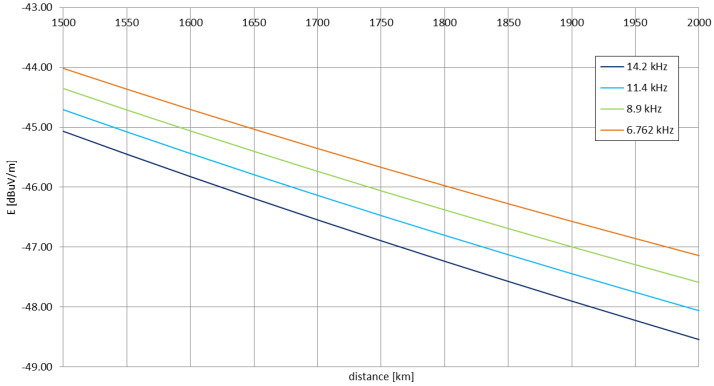
The propagation curves of the considered VLF signal from the altitude-gaining emitter, calculated using the Austin-Cohen formula [52] for the larger distances and radiated power of 1 μW. The general tendency of the E rising with the decrease of the frequency (the increase of the emitter’s altitude) can be noticed, yet with no characteristic oscillations typical for the VLF propagation. For shorter distances, the curves tend to converge to a single curve. For the LMP simulation, such convergence starts to manifest itself below the distances of 200 km only).

As the altitude increases in the simulation, the oscillations of the signal’s functions decrease in amplitude. For the distances below 1000 km, the registered points for the maximum altitude (for the lower altitudes, the signal in these locations has either vanished or decreased in strength) have appeared between the major null and the local maximum after it. Their logarithmic interpolation is, therefore, purely visual, similar to the approach for the definition of the Austin-Cohen formula. The electric field strengths for the same distances and frequencies can be calculated using this formula (shown in Figure 15), yet no complexity of the LMP simulations, nor the behaviors from the measurements can be noticed.

The short registration of the signal in the moment of the incidental contact of the antenna with the anemometric mast during the landing corresponds to the ground case of 14.2 kHz in Figure 14. Yet, as the antenna started to function as a different type of radiator (with extensive ground and underground installations) than the fully airborne one and the simulated one (antenna element), it cannot be directly compared to the rest of the experimental results and the simulations. The signal recorded at 1750 km from the receiver (Guingamp, France) does not comply with the patterns from the simulations, either because of the possible local signal maximum (sea proximity) or an error in the registration (low FFT quality).

In order to fully describe and compare the actual propagation path of the emitted signal with the performed simulations, a larger number of measurement points close to the locations of the moving major null and local maximum after it should be implemented. More detailed and less generalized simulations could be delivered using more detailed signal propagation path ground data, as in [19], where military data on the path were employed. Past (classic) simulations and experiments have shown a clear dependence of the signal strength on its frequency on equal propagation path [5,61], with a similar behavior observed for the increase of altitude of the emitter of various polarizations [17]. If these phenomena are compounded using the modal propagation theory from Wait [23] (Section 2.3 and Figure 4), namely the receiver height (altitude) and its corresponding frequency, an apparent (indicating the change of propagation behavior and dominating mode) decrease of the transmitting frequency with the rising altitude can be stated. The LMP simulation based on this statement is generally consistent with the experimental measurements for a physically rising vertical long wire VLF transmitting system operating (electronically) on a constant frequency. More detailed and less general comparisons with the simulated propagation behavior would be possible after multiple VLF transmitting missions (preferably spanning through different times of the year/seasons).

The effect of the evolving radiation pattern of the balloon-lifted antenna requires further analysis, e.g., incorporating in (3)–(5) and (7) the directions/angles of the increased power emission. A moderate effect of the maximum directivity of the antenna reached at the altitude of approximately 9.7 km could be traced on the spectrum in Figure 9, but this particular phenomenon requires greater insight in order to be fully confirmed and to have its actual influence analyzed.

### 7.2. Comparison with Low Frequency Experiments

The apparent frequency decrease for a lower-frequency radio emitter elevated at a substantial altitude have also been observed in [54], where a most convenient ground-positioned antenna was tested—an HML (horizontal magnetic loop) positioned on the top of the Klein Matterhorn Mountain (~3.9 km above the mean sea level) in Switzerland, operating as an inductive device at the center frequency of 270 kHz within the 9 kHz bandwidth. Apart from the specific formula describing the propagation of such a signal (adapting the popular ‘sum-of-square-roots’ formula by Vviedenskiy [52]), a dependence on the frequency has been found in the propagation curves provided for different frequencies by the CCIR—an equally achieved range from a ground-based vertical-antenna emitter has been reported for the 2× lower frequency than the one actually used in the HML tests on Klein Matterhorn.

**Figure 16:**
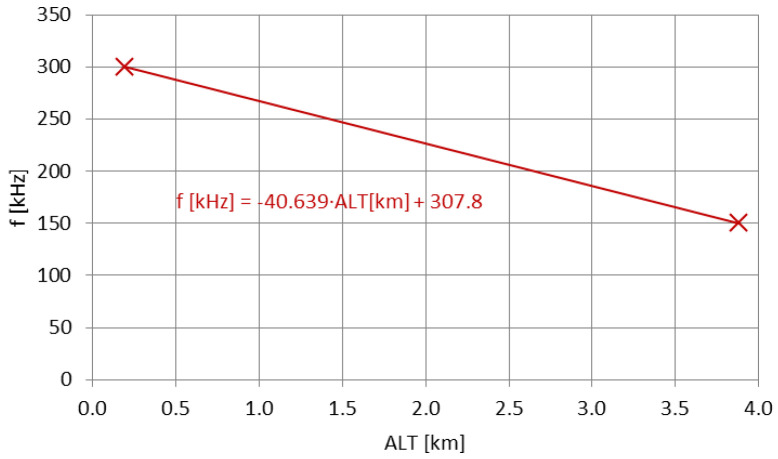
The apparent frequency decrease prediction function plotted for the LF/270 kHz case from [54]. The formula is based on the maximum obtainable signal coverage from the HML elevated antenna in reference to the ground-based antenna from RKS Topolná (the main transmitting site on 270 kHz [62]).

**Figure 17:**
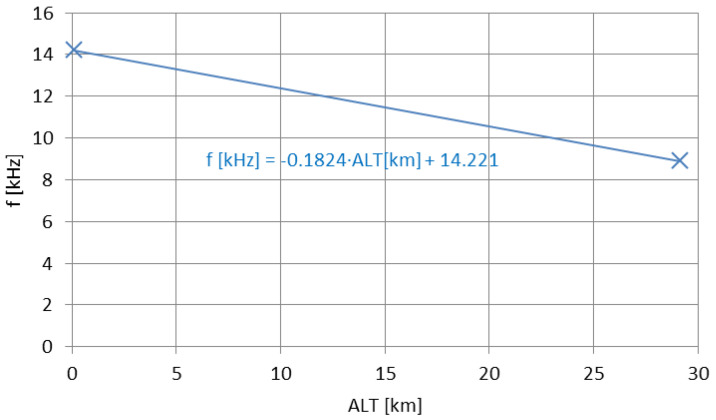
The apparent frequency decrease prediction function plotted for the VLF/14.2 kHz case of a fully airborne light-balloon experiment.

The apparent decreases of the frequencies for both cases (airborne-VLF and mountain-LF) have been plotted in Figure 16 and Figure 17. Figure 16 employs a comparison with the main transmitting site on 270 kHz, the RKS Topolná in Czechia [62]; therefore, the formula shown in this plot is valid for the transition from a ground-plane vertical antenna to an HML antenna above the ground. The difference in the signal coverage between the same type of antenna, i.e., the HMLs positioned on the mountain and on the ground, is natural. Tests conducted in 2021 with the employment of the HML of the parameters analyzed in [63], showed the decrease of the emitted signal on 198 kHz (signal type: A3E-SC, modulation index same as in [54], max. electrical power consumed: 15 mW) below 2.5 mW/m 50 m away from the HML’s center, with no external reports received. Even for the augmentation of the transmitter’s power to match exactly the level reached in [54] (but for the same modulation index), the ground-based HML in this case is not expected to surpass the mountain-elevated HML in signal coverage.

Both prediction functions in Figure 16 and Figure 17 are linear; this has been employed consistently with the equally linear frequency decrease mechanism described in Section 2.3. The differences between the two functions depend on the antennas used and the operating frequency (hence the different slope factors of the functions), yet the general behavior remains the same. Therefore, it may be stated that the apparent frequency decrease with the increasing altitude of the emitter is a general property in the terrestrial waveguide, yet its confirmation and overall accuracy requires more data to be analyzed for different types of transmitting antennas and different wavelengths (in order to define, e.g., the lower and the upper frequency limit of this property).

### 7.3. Possible Ameliorations and System Employment

A fully airborne VLF antenna system appears as a more efficient tool for VLF emission in comparison with systems incorporating the aircraft-trailed antennas. Ddespite using a different mode of transportation, it is able to successfully cover a substantial range with a detectable signal. The stratospheric balloon, apart from employing more efficient mode excitations and simpler mode composition, allows the transmitter to be moved to the part of the planet’s atmosphere where the only practical way to interfere with such a system is by using rocket-type objects able to reach high altitudes, which substantially increases the safety of such installation in comparison to a terrestrial application. The use of such fully airborne VLF transmitting systems could employ the support for the existing terrestrial VLF stations, the manipulation of signal coverage of hostile emissions (by, e.g., the modification of positioning of their signals’ major nulls) and atmospheric remote sensing—e.g., the monitoring of the ionosphere’s state (electron density, layer heights, ionospheric scattering etc.).

The shown experiment has been implemented in a light-balloon mission in a repetitive form, and it is easy to launch in different weather conditions. This demands certain compromises in design in order to mitigate the risks associated with the mission while remaining compliant with the obligatory legal documents. With the increase of the mission’s weight class (with the maintenance of the latex balloon type), along with the employment of legally required navigational and operational equipment and procedures, numerous ameliorations to the design of the experiment could be implemented. For instance, a more powerful transmitter (with a GPS-tuned frequency generator and a preferable power amplifier type transition to the D-class (analogous e.g., to LW broadcasting systems [64], reducing heat losses and reducing the risk of possible overheating in the stratosphere), more efficient antenna transformer (which demands more payload margin), a system for the automatic overload detection for the power amplifier in order to actively protect the transmitter from the currents from electrical discharges (this also demands more payload margin), and finally, greater lengths of the radiating wire, increasing the radiation resistance and the efficiency of the transmission. Even further improvements could concentrate on the antenna itself by using either passive or active lift-generating surfaces positioned on the wire, the antenna could twist its spatial shape in resemblance to a circularly polarized monopole. This could induce more compounds in the modal propagation of the signal, altering the signal coverage over distances shorter than 500 km. An open question is the accurate definition of the actual antenna’s parameters, as the antenna, due to its dimensions, remains difficult to measure on the ground and passes through a significantly changing environment. Some of the atmospheric features, e.g., the temperature inversion and large cloud layers/storm fronts, could modify the functioning of the antenna and the behavior of the emitted signal, adding additional factors to the modal propagation of the airborne VLF.

For the described experiment, a simple carrier-wave analogue transmission type was employed, however digital transmission types developed for low-frequency and low-bandwidth communication could bring an amelioration to the effective information decoding over large distances. A mission employing an WSPR digital signal exciter substituting the previous frequency generator was prepared and launched on 18th December 2021 with the same repetitive experimental setup [33], yet the mission did not deliver any substantial data, as the exciter’s lithium battery failed at higher altitudes, putting out the transmitter (while the pre-flight bench tests of the transmitter proved successful). As the launch location chosen for this mission was assigned appropriately, the antenna system was deployed completely and correctly, with the landing on the trees with the antenna in the correct, elongated position. The entire experiment—in the new, payload-augmented experiment, or in the previous, light formula—could be repeated in the future in order to deliver higher amounts of data to validate the described propagation mechanisms beyond generalities, towards a higher level of accuracy and detail.

## 8. Conclusions

An unanchored, stratospheric, light-balloon experimental mission employing a VLF transmitter equipped with a vertical antenna has been prepared and analyzed in the terms of the risks associated with mechanical, thermal, electrical, and operational aspects of utilization. Based on the outcomes of previous passive fully airborne VLF antenna experiments, as well as other stratospheric missions (both domestic and international), solutions providing an acceptable level of safety of such a balloon mission have been developed and included in the physical design of the actual experiment, the performance of which was theoretically estimated as superior in comparison with ground-based emitters. The experiment was successfully flown in 2020 from the EPPZ airport in Poland, reaching a maximum altitude of 29,164 m above mean sea level and transmitting on 14.2 kHz with low power. The obtained reception reports from different European countries have shown the changes in the signal’s coverage remaining in general accordance with the performed simulations, and based on the developed hypothesis of the apparent frequency decrease with the rise of altitude of the VLF emitter (with a similar phenomenon previously indicated for low frequency mountain experiments). The theory upon which the hypothesis was formed could be, therefore, employed for the general prediction of signal coverages by airborne VLF vertical emitters by the use of ground-based emitters with lowered operating frequencies. Additional data on this phenomenon, as well as the performance of a fully airborne VLF vertical transmitter, could be delivered by the expansion and possible unification of the reception network, as well as the introduction of more sophisticated transmitter subsystems and digital signal transmissions for future experimental missions planned for different parts of the year, in order to deliver more substantial data on the actual behavior of the described propagation mechanisms.

## Figures and Tables

**Table 1:** The severity and probability weights used for the risk assessments.

Severity		Probability	
1	No/minor damage	1	Extremely low
2	Damage not affecting performance	2	Low
3	Loss of performance	3	Medium
4	Subsystem shutdown	4	High
5	Mission destruction	5	Very high

## Data Availability

Not applicable.
